# Amylose rénale compliquant une maladie de Still de l’adulte: à propos de 3 cas

**DOI:** 10.11604/pamj.2019.32.158.6285

**Published:** 2019-04-08

**Authors:** Marzouk Sameh, Ben Salah Raida, Cherif Yosra, Jallouli Moez, Garbaa Saida, Kammoun Khawla, Hachicha Jamil, Bahloul Zouhir

**Affiliations:** 1 Service de Médecine Interne, CHU Hédi Chaker, Sfax, Tunisie; 2 Service de Néphrologie, CHU Hédi Chaker, Sfax, Tunisie

**Keywords:** Maladie de Still de l´adulte, amylose renale, inflammation chronique, Adult-onset Still’s disease, renal amyloidosis, chronic inflammation

## Abstract

L’amylose rénale est une complication rare au cours de la maladie de Still de l’adulte. Nous rapportons 3 observations d’amylose rénale parmi une série de 33 cas de maladie de Still de l’adulte. Il s’agit de 3 patientes d’âge moyen de 43 ans (extrême: 33 et 58 ans). Le diagnostic de maladie de Still a été retenu devant une polyarthrite fébrile (3 cas) associée à une éruption cutanée fugace (1 cas), un syndrome inflammatoire biologique en l’absence de toute cause infectieuse, inflammatoire ou néoplasique. Toutes ont été traitées par une corticothérapie associée secondairement au méthotrexate devant une polyarthrite destructrice (2 cas) et devant une récidive (1 cas). Une amylose rénale était survenue 4,9 ans après la maladie de Still (extrême: 33 mois et 7ans). L’amylose était révélée par un syndrome néphrotique (3 cas), associé à une insuffisance rénale (1 cas). Le diagnostic a été posé par une ponction biopsie rénale (3 cas) concluant à une amylose AA (2 cas) et non typée (1 cas). Tous les malades ont reçu la colchicine. L’évolution était favorable chez une patiente alors que les deux autres ont évolué vers l’insuffisance rénale chronique. L’amylose rénale est une atteinte inhabituelle de la maladie de Still de l’adulte. Une fois installée, elle peut engager le pronostic vital.

## Abstract (en)

Renal amyloidosis is a rare complication of adult onset Still’s disease. We here report three cases of renal amyloidosis in a series of 33 cases of adult onset Still’s disease. The three patients enrolled had an average age of 43 years (with a range from 33 to 58 years). The diagnosis of Still’s disease was retained on the basis of febrile polyarthritis (3 cases) associated with fleeting rush (1 case), biologic inflammatory syndrome in the absence of any infectious, inflammatory or neoplastic causes. All patients were treated with corticosteroids secondarily associated with methotrexate due to destructive polyarthritis (2 cases) and to a recurrence (1 case). Renal amyloidosis had occurred 4.9 years after Still’s disease (with a range from 33 months to 7 years). Amyloidosis was revealed by nephrotic syndrome (3 cases) associated with renal failure (1 case). Diagnosis was based on renal puncture biopsy (3 cases) which showed AA amyloidosis (2 cases) and untyped amyloidosis (1 case). All patients received colchicine. Outcome was favorable in a female patient while in the other two patients the disease progressed to chronic renal failure. Renal amyloidosis uncommonly results from adult onset Still’s disease. Once the disease gets established it can be life-threatening.

## Introduction

La néphropathie amyloïde reste une pathologie fréquente en Tunisie, compliquant le plus souvent les maladies infectieuses chroniques, en particulier la tuberculose pulmonaire, les rhumatismes inflammatoires chroniques, la fièvre méditerranéenne familiale et certaines néoplasies [1]. Elle est exceptionnelle au cours de la maladie de Still de l’adulte (MSA) et met alors en jeu le pronostic vital [2]. Depuis sa première description par Harrington *et al*. en 1981, plus d’une vingtaine d’observations ont été décrites [3]. Nous rapportons 3 nouvelles observations d’amylose rénale survenues au décours évolutif de MSA parmi une série de 33 cas.

## Patient et observation

### Observation n°1

Madame G.F. âgée de 58 ans, était hospitalisée en mai 1993 au Service de Médecine Interne pour une polyarthrite chronique des grosses articulations dans un contexte d’altération de l’état général, une fièvre à 38,5°C, des myalgies et une éruption cutanée fugace des zones découvertes. La biologie montrait un syndrome inflammatoire avec une vitesse de sédimentation (VS) à 135mm à la première heure, une fibrinémie à 24g/l, une hyperleucocytose à 16300 elts/mm^3^ et une anémie inflammatoire à 6g/dl. L’enquête infectieuse était négative (sérologies virales, bactériennes, parasitaires, hémocultures, échographie cardiaque…). La biopsie de l’artère temporale était normale. Le myélogramme et la biopsie ostéomedullaire étaient normaux. La sérologie rhumatoïde (SR) et les anticorps antinucléaires (AAN) étaient négatifs. L’échographie abdominale montrait une splénomégalie homogène sans adénopathies profondes. Les radiographies articulaires et du thorax étaient normales. Le diagnostic de MSA était retenu et une corticothérapie à 1mg/kg/j était prescrite avec dégression progressive entraînant une amélioration clinique et biologique. En février 1998, on associait le méthotrexate à une dose hebdomadaire de 10mg/semaine devant l’installation d’une polyarthrite destructrice. En mai 1998, on découvrait un syndrome néphrotique avec une protéinurie à 7,7g/24H sans hématurie ni leucocyturie ni insuffisance rénale et sans hypertension artérielle (HTA). La ponction biopsie rénale (PBR) a conclu à une amylose rénale. Le méthotrexate était maintenue associée au prednisone (10mg/j), à un anti-agrégant plaquettaire et à la colchicine (1mg/j). Une analyse moléculaire, découvrait l’existence d’une mutation E148Q à l’état homozygote et confirmant le diagnostic d’une maladie périodique associée. L’évolution était marquée par la persistance de la protéinurie à 7g/24H et l’apparition d’une insuffisance rénale (créatinine à 220μmol/l). En janvier 2000, la patiente présentait une gastro-entérite fébrile, responsable d’une décompensation rénale. Elle était décédée par un état de choc septique.

### Observation n°2

Madame S.K. âgée de 38 ans, sans antécédents pathologiques particuliers, était hospitalisée en juin 1996 pour une polyarthrite chronique dans un contexte d’altération de l’état général et d’une fièvre vespérale. La biologie montrait un syndrome inflammatoire biologique avec une VS à 138mm à la première heure, une anémie inflammatoire à 8,8g/dl et une hyperleucocytose à 10900elts/mm^3^ dont 85% de polynucléaires neutrophiles. La fonction rénale était normale. La protéinurie de 24H était négative. Il n’y avait pas d’anomalies des sédiments urinaires. Le bilan hépatique montrait une discrète cholestase à une fois et demi la normale. L’enquête infectieuse était négative. Les anticorps antinucléaires AAN, la SR et la cryoglobulinémie étaient négatifs. La radiographie articulaire, du thorax et l’échographie abdominale étaient normales. L’échographie cardiaque montrait une péricardite de faible abondance. Le myélogramme et la biopsie ostéo-medullaire étaient normaux. La ponction biopsie hépatique montrait une stéatose hépatique. Le diagnostic de MSA a été retenu et une corticothérapie à 1mg/kg/j a entraîné une amélioration clinico-biologique. En juillet 1997, on associait le méthotrexate à la dose de 10mg/semaine et des anti-inflammatoires non stéroïdiens devant l’installation d’une polyarthrite destructrice. En juin 2003, la patiente présentait un syndrome néphrotique avec une protéinurie à 5,4g/24H associée à une insuffisance rénale (créatinine à 147μmol/l avec une clairance de la créatinine à 27ml/mn). Il n’existait pas d’hématurie. La PBR a conclu à une amylose de type AA. On diminuait les anti-inflammatoire non stéroïdien (AINS) et on maintenait le méthotrexate et le prednisone à la dose de 10mg/j. En avril 2004, la patiente a aggravé son insuffisance rénale (une clairance à 9 ml/mn), nécessitant ainsi une hémodialyse chronique.

### Observation n°3

M^lle^ HOA âgée de 33 ans, sans antécédents pathologiques particuliers, était hospitalisée en août 2000 au Service de Médecine Interne pour une polyarthrite fébrile évoluant depuis 3 mois. L’examen clinique trouvait une fièvre hectique à 39°C et une arthrite touchant les 2 poignets et les 2 genoux. La biologie montrait une VS à 100mm à la 1^ère^ heure, une fibrinémie à 7,3g/l, une hyperleucocytose à 13400elts/mm^3^ dont 85% de polynucléaires neutrophiles et une anémie inflammatoire à 9,6g/dl. La ferritinémie était augmentée à 1445ng/ml. La fonction rénale était normale. La protéinurie de 24 heures était négative. Le sédiment urinaire n’a pas montré d’hématurie. Il existait une choléstase et une cytolyse hépatique. L’enquête infectieuse était négative. Le FR, les AAN, les anti-muscles lisses, les antimitochondriaux et la cryoglobulinémie étaient négatifs. Les radiographies articulaires et du thorax, l’échographie abdominale et cardiaque étaient normales. La ponction sternale était normale. La ponction biopsie hépatique montrait une inflammation non spécifique. Le diagnostic de MSA était retenu et la patiente a été mise sous corticothérapie à 1mg/kg/j entraînant une régression de la fièvre, des signes articulaires et du syndrome inflammatoire. En février 2001, elle présentait la même symptomatologie clinique nécessitant la reprise des corticoïdes et l’adjonction du méthotrexate à la dose de 10mg/semaine entrainant une bonne évolution. En mai 2003, elle était réhospitalisée pour des œdèmes des deux membres inférieurs et du visage. La tension artérielle était à100/60 mmHg. Il n’existait pas de poussée articulaire ni de macroglossie. A la biologie, on trouvait un syndrome néphrotique avec une protéinurie à 8g/24H, une hypo albuminémie à 11g/l et une hypoprotidémie à 44g/l. La fonction rénale était normale (75μmol/l), il n’existe pas d’hématurie ni de leucocyturie. La PBR a permis d’analyser 28 glomérules sans pain à cacheter et a conclu à une amylose. L’immunofluorescence directe était positive pour l’anticorps anti-amyloïde A. Le Méthotrexate était maintenu à une dose hebdomadaire 10 mg, associé à 10 mg/jour de Prednisone. On a associé la Colchicine à la dose de 1mg/jour, un anti-protéinurique et un anti-vitamine K (AVK). En décembre 2004, la patiente était en rémission clinique de sa maladie de Still et on a arrêté le méthotrexate et les AVK. Depuis 2005, elle a annulé la protéinurie. En 2006 et 2010, la patiente a mené 2 grossesses sans aucune complication materno-foetale. Le recul actuel est de 7 ans.

## Discussion

La maladie de Still est une maladie systémique inflammatoire de cause inconnue. En 1971, elle a été en fait décrite pour la première fois chez l'adulte. Le diagnostic est difficile et repose à la fois sur des critères cliniques et biologiques. C'est avant tout un diagnostic d'exclusion de maladies virales, bactériennes, hématologiques ou auto-immunes. Nos 3 patientes présentent une MSA devant l’association de critères majeurs et de critères mineurs de Yamaguchi [4]. La survenue de l’amylose au cours de l’évolution de cette maladie systémique, en l’absence d’une pathologie infectieuse, inflammatoire ou tumorale permet de la rattacher à la MSA. Notre deuxième malade présente également une maladie périodique de découverte fortuite sans traduction clinique, qui peut se compliquer elle-même d’une amylose rénale. Chez cette même malade l’amylose n’a pu être typée.

L’amylose inflammatoire ou amylose AA reste une complication de l’inflammation chronique, bien que son incidence diminue dans les pays occidentaux et probablement dans d’autres pays comme la Tunisie. Au cours des 30 dernières années, une meilleure maîtrise de l’inflammation qu’elle soit d’origine infectieuse, tumorale, génétique ou liée à une maladie inflammatoire explique probablement la diminution de l’incidence de l’amylose AA. La prévalence de l’amylose AA au cours de la polyarthrite rhumatoïde s’échelonne ainsi de 3 à 23% alors qu’elle est de 5% au cours de la spondylarthrite ankylosante [5].

La fréquence de survenue de l’amylose au cours de la MSA est encore inconnue. Depuis sa première description par Harrington *et al.* en 1981 [3], plus d’une vingtaine d’observations ont été décrites, soit sous la forme d’observation unique [6-8], soit faisant partie de séries [9, 10]. Dans notre série de 33 cas de MSA, la fréquence de l’amylose rénale est de 9%. La pathogénie de l’amylose au cours de la MSA demeure hypothétique. L’élévation chronique de la « sérum amyloid associated protein » (SAA sérique) est le facteur essentiel qui contribue à la formation de l’amylose au cours des maladies inflammatoires chroniques [11, 12]. Cependant, tous les malades ayant une maladie inflammatoire chronique et une augmentation prolongée de la SAA sérique ne développent pas d’amylose. Il existe donc des facteurs supplémentaires génétiques et environnementaux qui favorisent cette complication [13]. Le délai de survenue de l’amylose par rapport à la MSA varie de 18 mois à 40 ans, soit une médiane de 9 ans [8]. Elle était de 4,9 ans en moyenne chez nos patientes. L’amylose complique souvent les formes polyarticulaires de MSA qui évoluent vers l’ankylose et la destruction radiologique. Deux de nos patientes ont évolué vers une polyarthrite destructrice. Chez ces deux patientes, l’amylose est survenue après 5 ans (observation n°1) et 7 ans (observation n°2).

Dans les cas rapportés, l’amylose est le plus souvent rénale, s’exprimant par une protéinurie ou un syndrome néphrotique. L’atteinte digestive se manifeste par une diarrhée motrice ou une pseudo-occlusion intestinale. Le diagnostic de l’amylose reste histologique et repose sur les résultats de la PBR, de la biopsie rectale ou de la graisse abdominale [8]. Compte tenu du caractère diffus des dépôts amyloïdes et du caractère invasif de la biopsie rénale, la tendance actuelle est de pratiquer en première intention des biopsies de sites plus accessibles telles les glandes salivaires accessoires dont la sensibilité varie de 86 à 100% [14]. La néphropathie amyloïde évolue naturellement vers l’insuffisance rénale chronique [8, 10]. Elle peut se compliquer de thrombose veineuse, notamment des veines rénales, d’insuffisance rénale aiguë et le syndrome néphrotique peut persister alors que l’insuffisance rénale est avancée, avec un risque accru de pertes protéiques et de dénutrition [5].

Dans notre série, elle était annoncée par un syndrome néphrotique pur (2 cas) et impur avec insuffisance rénale (1 cas). Le recours à l’hémodialyse a été indispensable chez une patiente après 18 mois d’évolution. Le traitement de l’amylose secondaire au cours de MSA comprend plusieurs aspects et étapes. L’amylose doit être combattue en renforçant le traitement anti-inflammatoire, car l’évolution semble directement liée à la maîtrise de l’inflammation et de la concentration sérique de la protéine SAA. Le traitement préventif par de la colchicine dans les formes non contrôlées de la MSA prévient autant les poussés inflammatoires que l’amylose qui en est la conséquence mais aucun examen biologique ne permet d’en affirmer son efficacité [5, 7]. Une fois l’amylose installée, une réduction de la disponibilité du précurseur de la protéine amyloïde est actuellement l’approche thérapeutique la plus logique pour toutes les formes d’amylose avec l’objectif théorique d’arrêter la progression des dépôts. Ce concept doit conduire à abaisser autant que possible la concentration sérique de protéine SAA ou de la protéine C réactive quand le dosage de la SAA n’est pas disponible [5].

Cependant, les données acquises sur l’efficacité respective des différents médicaments les plus efficaces pour traiter l’amylose inflammatoire restent modestes. Plusieurs études suggèrent l’intérêt du chlorambucil et du cyclophosphamide dans cette indication [8, 15, 16]. De nouveaux traitements ont transformé la prise en charge de plusieurs maladies inflammatoires chroniques. A l’heure actuelle, on ne dispose que de quelques études rapportant des cas isolés ou de courtes séries de malades atteints d’amylose compliquant des maladies inflammatoires chroniques et traités par étanercept et/ou infliximab ou tocizulimab, souvent associés à d’autres médicaments immunosuppresseurs [11, 16-19]. Les réponses sont toujours évaluées à court terme. Les résultats peuvent être considérés comme encourageants, toujours spectaculaires dans les cas isolés [18, 20], beaucoup moins convaincants dans les séries même courtes, ou certains malades ne répondent pas au traitement. Ces résultats plutôt favorables sont à mettre en balance avec les effets secondaires graves, en particulier les infections [16, 19].

## Conclusion

L’amylose rénale au cours de la maladie de Still de l’adulte est une complication rare. Elle est de 9% dans notre série. Une fois installée, son pronostic reste préoccupant. Des progrès thérapeutiques sont nécessaires, en particulier la mise au point de médicaments ciblés sur les phases intimes de l’amylogenèse.

## Conflits d’intérêts

Les auteurs ne déclarent aucun conflit d'intérêts.
